# Synthesis of a mesoporous titania thin film with a pseudo-single-crystal framework by liquid-phase epitaxial growth, and enhancement of photocatalytic activity†

**DOI:** 10.1039/d0ra08019e

**Published:** 2020-11-09

**Authors:** Norihiro Suzuki, Chiaki Terashima, Kazuya Nakata, Ken-ichi Katsumata, Akira Fujishima

**Affiliations:** Photocatalysis International Research Center, Research Institute for Science and Technology, Tokyo University of Science 2641 Yamazaki Noda Chiba 278-8510 Japan suzuki.norihiro@rs.tus.ac.jp; Graduate School of Bio-Applications and Systems Engineering, Tokyo University of Agriculture and Technology 2-24-16 Nakacho Koganei Tokyo 184-0012 Japan; Faculty of Industrial Science and Technology, Tokyo University of Science 6-3-1, Niijyuku Katsushika Tokyo 125-8585 Japan

## Abstract

A mesoporous titania thin film with a pseudo-single-crystal framework was synthesized on a lanthanum aluminate single-crystal substrate by a surfactant-assisted sol–gel method and liquid-phase epitaxial growth. The crystal lattices were well aligned within the titania framework. The highly energetic {001} facet was exposed on the top surface, which significantly enhanced the photocatalytic activity.

## Abstract 2

An anatase-phase mesoporous titania thin film with a pseudo-single-crystal framework was facilely synthesized by an inexpensive chemical process.

## Graphical abstract

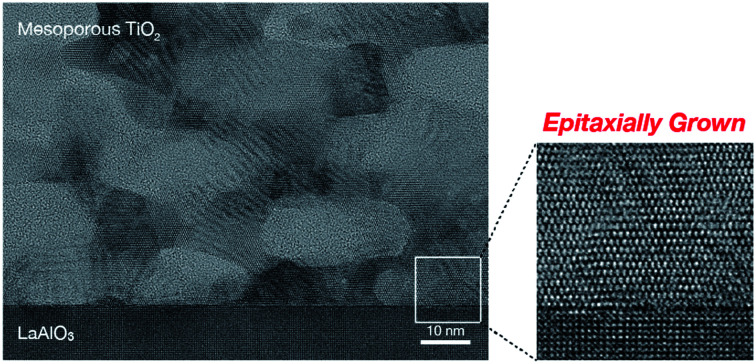

## 1. Introduction

Mesoporous materials with pores of diameter 2–50 nm have properties such as a large pore volume, high surface area, and uniform mesopore size. Mesoporous materials therefore have a range of potential applications, *e.g.*, as catalysts, catalyst supports, adsorbents, and molecular sieves. Surfactant-assisted sol–gel methods, in which self-assembled amphipathic surfactant micelles are used as an organic template, enable facile synthesis of mesoporous materials. Starting from mesoporous silica,^1^ various mesoporous metal oxides, *e.g.*, titania and alumina, were first synthesized in 1998.^2^ Unlike silica, which is insulating and chemically inert, metal oxides have various useful physical properties. Mesoporous metal oxides are therefore attractive in terms of practical applications.

Generally, crystallites in the framework of a mesoporous thin film synthesized *via* a surfactant-assisted sol–gel method are randomly oriented.^3^ However, if the targeted physical property of the framework material originates from crystal lattice anisotropy, randomly oriented crystallites weaken this property. Control of the orientation of crystallites is therefore a key factor in making full use of mesoporous materials.

Epitaxial growth is used to fabricate thin films with controlled lattice orientations. By selecting a proper substrate of which lattice parameter is quite similar to that of a grown target, the orientation of a grown layer is guided by the crystal face of the single-crystal substrate. Synthesis of the epitaxial layer usually involves complicated and expensive methods such as molecular beam epitaxy and chemical vapor deposition. However, for practical applications, simpler and inexpensive fabrication methods are preferable. The use of liquid-phase epitaxial growth and a sol–gel process is therefore attractive.

Several previous studies of thin-film synthesis by in-liquid epitaxial growth have been reported. Ferroelectric materials are major targets because anisotropy enhancement significantly improves polarization and piezoelectric coefficients. Sol–gel-derived epitaxial films of BaTiO_3_,^4^ (Ba,Sr)TiO_3_,^5^ PbTiO_3_,^6^ Pb(Zr,Ti)O_3_,^7^ Pb(Nb,Zr,Ti)O_3_,^8^ (Pb,La)(Zr,Ti)O_3_,^9^ and BiScO_3_–PbTiO_3_ (ref. 10) have been reported. In addition, multiferroic BiFeO_3_ (ref. 11) and GaFeO_3_ (ref. 12) thin films, a (La,Ca)MnO_3_ thin film with high magnetoresistance,^13^ and some buffer layers (BaZrO_3_,^14^ LaAlO_3_,^15^ and MgO^16^) have been prepared.

In contrast, there are few reported studies of titania, although its photocatalytic efficiency depends on the facet orientation.^17^ Selvaraj *et al.* used a sol–gel method to fabricate epitaxially oriented titania thin films on a rutile (110) substrate,^18^ and Chen *et al.* reported hydrothermal epitaxy of a highly (112)-oriented anatase titania thin film on a Si (100) substrate.^19^ However, the photocatalytic activities were not examined in these studies. Jung *et al.* reported the synthesis of a sol–gel-grown epitaxial titania thin film on a LaAlO_3_ (001) substrate, which had strong photocatalytic activity because of its enhanced photocarrier mobility.^20^

To achieve the better photocatalytic activity, increasing both reaction sites and photo-generated carriers that can reach these sites are significant. Introducing the porosity brought not only a large surface area but also thinner titania framework, which facilitates photo-generated carriers to reach the surface before the recombination. Therefore, compared to the bulk (nonporous) titania thin film in the previous study,^20^ obtaining porous titania thin film is much important for enhancing the photocatalytic activity. But, to the best of our knowledge, the synthesis and photocatalytic activity of an epitaxially grown mesoporous titania thin film have not yet been reported.

In this paper, we report the synthesis of a titania thin film with a well-ordered mesoporous structure from a surfactant-containing titania sol on a lanthanum aluminate (LaAlO_3_; LAO) substrate, of which lattice constant is similar to that of anatase titania. A pseudo-single-crystal framework was obtained, which confirms successful liquid-phase epitaxial growth.

## 2. Experimental

### 2.1 Materials

The diblock copolymer PS(18000)-*b*-PEO(7500) was acquired from Polymer Source Inc. (Dorval, QC, Canada). Titanium chloride, concentrated hydrochloric acid (35–37 wt%), tetrahydrofuran, ethanol, oleic acid, and *n*-heptane were purchased from the Fujifilm Wako Pure Chemical Corporation (Osaka, Japan). These chemicals were used as obtained. The LaAlO_3_ (100) substrate (15 × 15 × 0.5 t mm) was obtained from the Shinkosha Co., Ltd. (Yokohama, Japan).

### 2.2 Synthesis of mesoporous titania thin film

A mesoporous titania thin film was prepared by a previously reported method.^21^ First, PS(18000)-*b*-PEO(7500) (50 mg) was dissolved in tetrahydrofuran (1.5 mL) at 40 °C. After cooling to room temperature, ethanol (500 μL) was added and the mixture was stirred for 20 min (solution A). Separately, titanium chloride (150 μL) was quickly added to concentrated hydrochloric acid (200 μL). The components were mixed until the yellow solid intermediate was completely dissolved and then distilled water (300 μL) was added (solution B). Solution B was stirred for 10 min and then added dropwise to solution A. The mixture was stirred for 30 min to prepare the precursor solution. The precursor solution was dropped onto the LaAlO_3_ (100) substrate.‡‡Although a LAO (100) substrate was used, because LAO is pseudo-cubic, LAO (100) is equivalent to LAO (001). In this article, we refer to LAO (001) for simplicity. Spin-coating was performed with a spin-coater (Opticoat MS-100, Mikasa Co., Ltd.) at 3000 rpm for 30 s. The as-prepared film was calcined in air at 400 °C for 1 h (ramp ratio: 1 °C min^−1^) with an electric furnace (FO100, Yamato Scientific Co., Ltd.). A film was also fabricated on a glass substrate as a reference. The porous structure was investigated by examining top-view SEM images (JSM-7600F, JEOL).

### 2.3 Crystallographic characterization

Cross-sectional TEM images (H-9500, Hitachi) were obtained at an accelerating voltage of 200 kV to observe the lattice fringes. Sample specimens were prepared by the focused ion beam method. Out-of-plane and φ-scan XRD patterns were recorded with a Smart Lab (Rigaku) instrument at 45 kV and 200 mA, using Cu Kα radiation (*λ* = 1.5406 Å).

### 2.4 Photocatalytic activity test

The photocatalytic activity of the synthesized mesoporous titania thin film was examined by performing decomposition of oleic acid. The synthesized mesoporous titania thin film was pre-treated by irradiation with 365 nm UV light at 2.0 mW cm^−2^ for 1 day to remove residual organic components. After UV irradiation, the mesoporous titania thin film was dipped in 0.5 vol% oleic acid solution diluted with *n*-heptane, and then withdrawn at 10 mm s^−1^, with a dip-coater (M300S, Asumi Giken, Ltd.) After dip-coating, the sample was heated at 70 °C for 15 min with a hot plate (ND-1, As One Co.) to dry the oleic acid layer. The sample was irradiated with 365 nm UV light at 1.0 mW cm^−2^ to induce photocatalytic decomposition of oleic acid. During the test, samples were taken at various times and the water-contact angle was measured with a contact angle meter (DM-501, Kyowa Interface Science Co., Ltd.)

## 3. Results and discussions

**Fig. 1:**
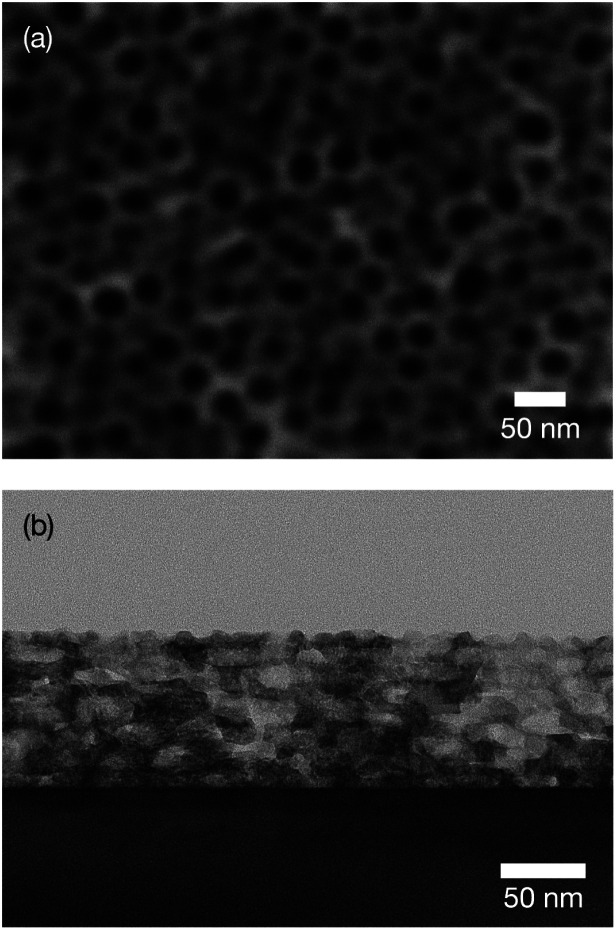
(a) Top-view SEM and (b) cross-sectional TEM images of obtained mesoporous titania thin film on LAO substrate.

**Fig. 2:**
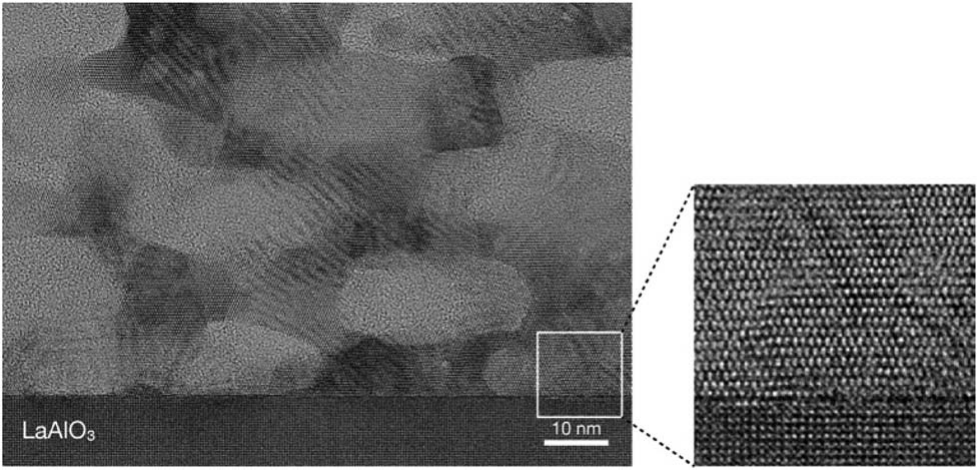
High-magnification cross-sectional TEM image of obtained mesoporous titania thin film.

Top-view SEM images and cross-sectional TEM images were used to examine the porous structure. As in a previous study, hexagonally packed mesopores of diameter around 20–30 nm were obtained [Fig. 1(A)].^21^ These mesopores were stacked throughout the entire film [Fig. 1(B)].^21*b*^ The film thickness was estimated to be around 100 nm. In a high-magnification image, lattice fringes were clearly observed around the mesopores (Fig. 2). Although the appearance of the lattice fringes of the crystallites sometimes differed slightly, depending on the thickness, defects, and curvature of the specimen, the orientation of the lattice fringe of each crystallite was basically parallel to the substrate. The magnified clearly showed ordered arrangements of atoms within the titania framework at the titania/LAO interface. It was therefore assumed that the entire film was epitaxially grown.

**Fig. 3:**
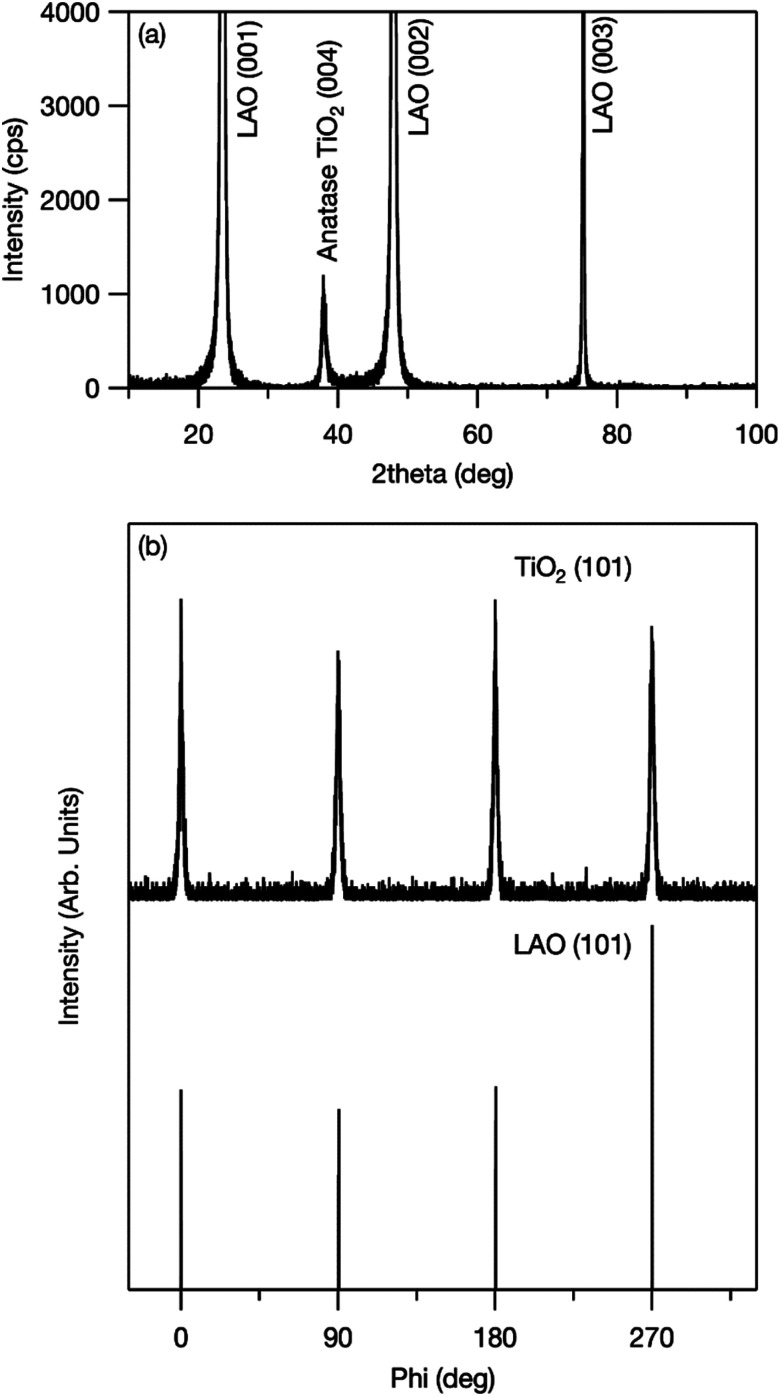
(a) Out-of-plane XRD pattern of mesoporous TiO_2_ thin film fabricated on LAO (001) single-crystal substrate and (b) XRD φ-scan results for TiO_2_ (101) and LAO (101).

An out-of-plane XRD pattern was recorded to examine this assumption. As shown in Fig. 3(A), only the anatase-phase (004) diffraction peak was observed. This indicates that titania was oriented to the *c*-axis (*i.e.*, the [001] direction) out of plane, and grown with TiO_2_ (001)‖LAO (001) in plane. In addition, to confirm the in-plane orientation, a φ-scan XRD pattern was recorded at the sample position at which TiO_2_ (101) and LAO (101) diffraction peaks were observed. If titania crystallites are aligned in the in-plane direction, four peaks from TiO_2_ (101) and from LAO (101) appear at the same angles. As shown in Fig. 3(B), both peaks appeared at 0°, 90°, 180°, and 270°, which shows in-plane alignment. These results confirm that the synthesized mesoporous TiO_2_ thin film was epitaxially grown on LAO and formed a pseudo-single crystal. The crystal structure of LAO is pseudo-cubic with a lattice constant *a* = 0.378 nm, whereas that of anatase titania is tetragonal and its *a*- and *c*-axis lattice parameters are 0.3785 and 0.9514 nm, respectively.^22^ The good match between the lattice parameters of LAO (001) and titania (001) provides a driving force for epitaxial growth.

**Fig. 4:**
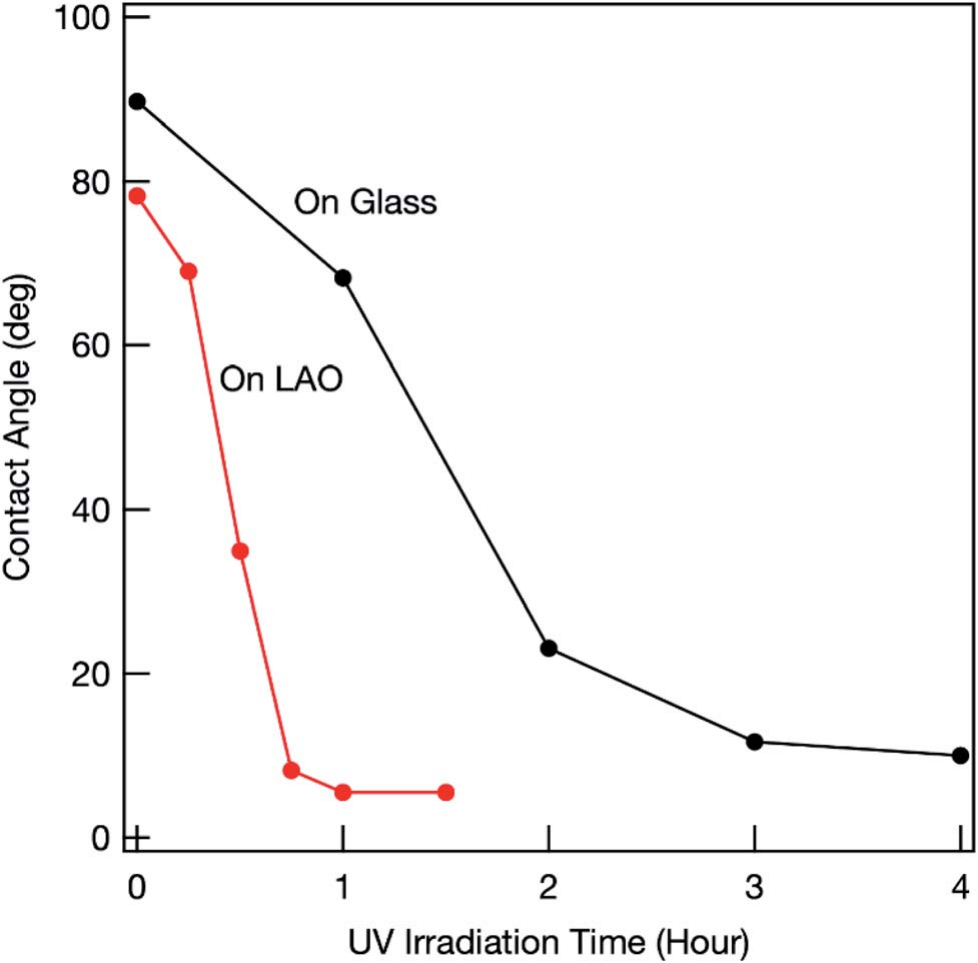
Changes in WCA for oleic acid-coated mesoporous TiO_2_ thin film under UV light irradiation.

The photocatalytic activity of the synthesized mesoporous titania thin film was examined by investigating the decomposition of oleic acid.^23^ As a reference, a film was fabricated on glass. Various anatase-phase diffraction peaks appeared in the grazing incident XRD pattern (Fig. S1†). This shows that the crystallites were randomly oriented when a glass substrate was used. Fig. 4 shows the changes in the water-contact angle (WCA) with UV irradiation time. Before UV irradiation, the WCA was relatively large (80°–90°), which shows that oleic acid was successfully coated on the surface. The WCA of the film fabricated on the LAO substrate decreased with increasing UV irradiation time because of photocatalytic decomposition of oleic acid, and reached around 10° or less (*i.e.*, the surface became super-hydrophilic) in around 1 h. This shows the superior photocatalytic activity of the film fabricated on LAO. Similar rapid decomposition of oleic acid was observed for another sample (Fig. S2†), which confirms that this superior photocatalytic activity is reproducible.

Previous studies showed that the photocatalytic activity of the highly energetic {001} facet is stronger than those of other energetically stable facets.^17^ Maitani *et al.* reported that the {001} facet facilitated photoexcited charge transfer from organic fluorophores to titania.^24^ This shows that photogenerated holes easily accumulated on the {001} facet. As explained above, the titania film obtained on LAO was *c*-axis oriented and the {001} facet was exposed on the top surface. The photogenerated holes within the film could easily move toward the top surface and the accumulated holes could effectively oxidize oleic acid.

In contrast, the decrease in the WCA was much slower when a glass substrate was used (Fig. S2†), which shows that the photocatalytic activity of the synthesized titania film was much weaker. Even for a well-fabricated film (Fig. 4), the photocatalytic activity was inferior to that of the film on LAO. Because there was no driving force for control, the crystal lattice was randomly oriented within the titania film fabricated on a glass substrate (Fig. S1†). Different facets therefore often contacted grain boundaries. Previous studies showed that photogenerated electrons mainly accumulate at the {101} facet.^25^ Photogenerated holes and electrons therefore recombine at the facet interface. The number of holes that can reach the top surface therefore decreases, which results in inferior photooxidation. The degree of photocarrier recombination differs among samples because of variations in facet orientation within the framework. This causes a lack of reproducibility (Fig. S2†).

## 4. Conclusions

A facile and inexpensive chemical process was used to synthesize an anatase-phase mesoporous titania (TiO_2_) thin film with a pseudo-single-crystal framework. Control of the crystal lattice orientation enabled full use of the photocatalytic activity inherent in the titania framework. This study will open up new methods for full utilization of functional mesoporous thin films.

## Conflicts of interest

There are no conflicts to declare.

## Supplementary Material

RA-010-D0RA08019E-s001
