# Supplementary material for: Synthesis of a mesoporous titania thin film with a pseudo-single-crystal framework by liquid-phase epitaxial growth, and enhancement of photocatalytic activity
Source: RSC Adv. 2020 Nov 9;10(67):40658–62. doi: 10.1039/d0ra08019e (PMC9057731; doi:10.1039/d0ra08019e)
Supplement: RA-010-D0RA08019E-s001 [file RA-010-D0RA08019E-s001.pdf]

Electronic Supplementary Material (ESI) for RSC Advances.
This journal is © The Royal Society of Chemistry 2020

Supplementary Information

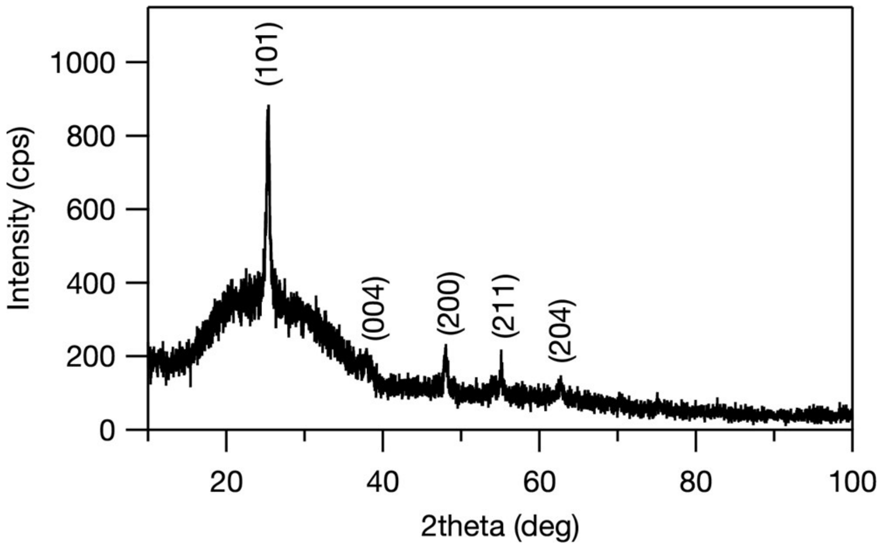

Figure S1. Grazing incident XRD pattern of mesoporous TiO_2 thin film fabricated on glass substrate. Assignments of crystal lattice indices for anatase phase are included.

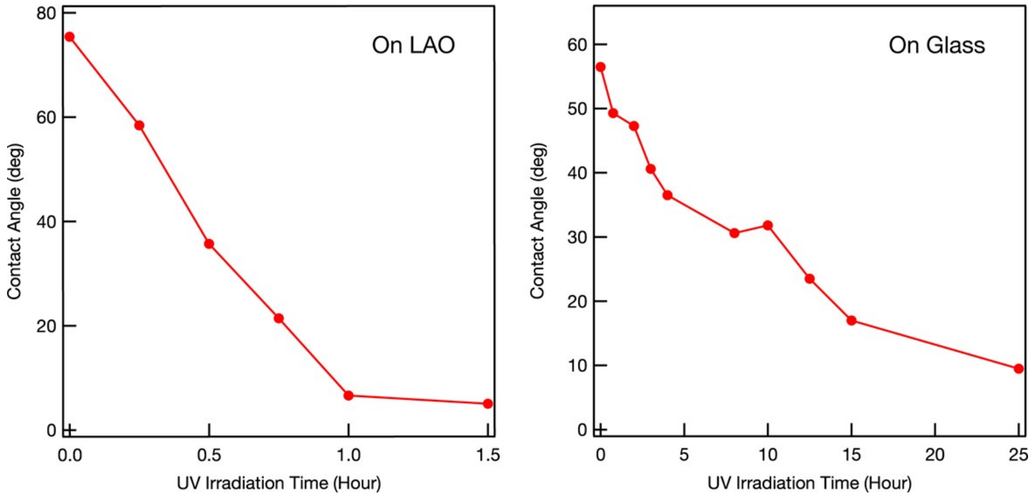

Figure S2. Changes in water-contact angle with UV irradiation time for oleic acid-coated mesoporous TiO_2 thin films fabricated on LAO (001) single-crystal and glass substrates.
